# Cystitis in the Female

**Published:** 1904-06

**Authors:** 


### CYSTITIS IN THE FEMALE.*

Cystitis in the female you find rather a common disease, both the acute and chronic forms. It can not help but be noted that in some it is a symptom of other trouble, yet comparatively little

*Read by Chas. W. Hibbitt, A.B., M.D., before the Society of Physicians and Surgeons Louisville, Ky.

notice of it will be taken by patients until a true cystitis has developed.

Amongst the predisposing causes we may mention vesical irritation by uterine displacements, tumors, pregnancy, and stone, all the above causing a difficulty in the passage of urine, resulting in retention and decomposition, followed by irritation and inflammatory changes in the mucous membrane, producing a suitable soil for the bacteria, and thus cystitis develops. Chemical changes in the urine resulting from certain diet or the use of irritating drugs may produce the conditions favorable for infection. Inflammation and infection are also produced by injuries from introduction of catheter, sound, or instruments of any kind.

Any infectious or inflammatory condition about the vulva or reproductive organs may extend to the urethra and bladder.

Any infection in the kidney may also produce the condition by the infected urine coming in contact with the bladder walls.

To sum up, infection is without doubt the most frequent cause of cystitis.

Prominent among the exciting causes must be mentioned the gonococci, tubercle bacillus, staph. and strept. pyogenes, and bacillus coli communis.

One word as to infection by catheter. This is no doubt caused by the nurse or physician trying to pass catheter by touch and not by sight, even without cleansing parts, thus forcing septic material from the external genitals into the urethra or bladder. With the foregoing facts, we can say cystitis is always caused by the presence of micro-organism where there is a predisposing cause.

It is necessary that we note the pathological changes, so we may arrive at a better diagnosis as to the variety. After irritation or infection the mucous membrane becomes congested and the light red or pink color gradually assumes a deeper and angry red, the epithelium is thrown off in some places, and here ulceration takes place and pus appears.

In cases where mixed infection is present, it will be difficult to distinguish between the different bacteriological varieties.

The diagnosis of cystitis is easily made by proper examination; we must, however, remember one point, that every female coming to us with history of vesical tenesmus, painful and frequent urination, does not necessarily have cystitis. These conditions are frequently caused by malpositions of the uterus, continued pressure of tumor, certain conditions of urethra, and also by cystocele following a relaxed perineum. These conditions, however, may and do produce cystitis by continuing for a period of time without being corrected.

It is, then, our duty to make a careful examination of the genital organs and others to exclude all such conditions which might produce this irritable bladder.

With the patient giving history of frequent urination, pain, alkaline urine and mucopus, there can hardly be any mistake in the diagnosis, but full knowledge as to its cause and variety will be ot very great value, and aid us materially in the treatment. The endoscope and cystoscope are of material aid to us in diagnosing the varieties, also the progress the subacute and chronic cases have made, as experience has taught us that direct inspection of the bladder wall yields the best results in the diagnosis.

The gonorrheal variety is usually an extension of the infection from the vulva and vagina up through the urethra, and needs only the appearance of the gonococci in the urine to clear up the cause.

So in the tubercular the diagnosis and cause is determined by the appearance of the tubercle bacilli in the urine, but they can not always be found present. The tubercle bacilli descending from the kidney to the bladder, this being the usual means of infection, my experience in tubercle cases which have come under my notice is that pain is not always present, and is not as severe as in other varieties.

Catarrhal cystitis, as a result ot a chronic inflammatory condition of the lining ot the bladder, is noted by the congested and reddened appearance of mucous membrane and the loosening of the epithelial cells.

The urine in this variety is usually acid, but may become alkaline if the case advances and does not improve; should this catarrhal condition not have prompt attention the inflammation may extend, involving the deeper structures of the bladder walls and produce a suppurative condition, the urine passed will contain large numbers of epithelial cells and pus.

The symptoms in the acute stage are frequent desire to pass water, and only a few drops at a time, with great burning pain along the urethra, and severe vesical pain also. An elevation of temperature which may be preceded by a chill, feeling of fullness and weight in region of bladder. The pain is constant, and is usually not relieved by emptying the bladder. We may have partial or complete retention of urine, or patient may pass small amounts frequently or it may dribble for a long time without emptying the bladder completely. In the chronic variety the symptoms are less severe than in the acute; pain is usually present at the beginning of act, and so makes the patient hold her urine longer; the urine, after being passed, becomes cloudy, and contains pus corpuscles, blood, mucus and uric acid crystals.

The constitutional condition of these chronic cases is worthy of note; they have no strength, no appetite, emaciation usually, sometimes chills and fever, and they gradually lose ground.

The prognosis is unually fair in the large number of cases, but considerable depends on the care and thoroughness of the treatment.

A few words as to the prevention of cystitis. Avoid the introduction of infectious material into the urethra and bladder. Avoid using catheter under bed-clothes, and without seeing what you are doing. Always have it sterile (which is an easy matter), and, in addition, disinfect the vulva, external meatus and parts about before passing.

Same conditions, due to faulty elimination, call for hygienic and medical treatment to correct this faulty condition, and thus prevent a concentrated chemically changed urine, which may affect the bladder walls and produce a cystitis. Any defect about the pelvis may obstruct the progress of labor, and the prolonged pressure upon the bladder may be the beginning of this trouble.

As to the treatment, first in the acute cases they must be put to bed. This in itself will give great relief; relieve pain and nervous irritability by morphine or opium in some form, but remember we must not keep the morphine up for any length of time, and we will have to resort to the ice or hot-water bag placed over the bladder and hot vaginal douches three or four times a day to relieve the congestion and to give our patient more comfort. In addition we may use the hot sitz baths. Laxatives are now indicated (salines by all means), given in small doses frequently (every few hours). Diet must be of the not stimulating variety. Milk by all means is preferable and indicated.

Drugs are indicated to change the quality and quantity of the urine. If the urine is excessively acid, citrate or acetate of potassium or bicarbonate of soda will give happy results, but always give with plenty of water. If the urine is alkaline, we should endeavor to make it mildly acid by the use of acids, benzoic or boric acid. Large quantities of alkaline water should be given—six, eight or ten glasses a day, to combat the acidity. In addition to the above, we may get some little effect from salol and boric acid every few hours, but to get any appreciable results from boric acid, it will have to be given in large quantities. Urotropin is also of value, but in my hands it has given much more brilliant results in the chronic than in the acute cases.

Hyoscyamus and belladonna must not be overlooked, because they certainly have a good effect on the vesical irritability, given internally or used in form of rectal suppository.

Do not consider using local treatment, namely, irrigation and application to the lining, during the acute stage.

If we find the trouble does not yield to the above treatment and it is passing into the subacute stage, we must adopt a different line. Now a more stimulating effect is desired upon the bladder walls, and we may use copaiba, oil eucalyptus and oil sandalwood; especially are these efficacious in the gonorrheal variety. Some few cases will have no severe symptoms whatever at the onset, and will be in the chronic state when they apply for treatment.

In the subacute and also the chronic stage is the local treatment to be used, that is, by applying to the diseased portion of the mucous membrane of the bladder through the endoscope and by irrigation through a catheter, allowing the solution to come in direct contact with the entire bladder wall.

Irrigation of the bladder is necessary; by it the bladder is cleared of large numbers of bacteria, and large quantities of mucus and pus, which would hardly be passed in the urine. This irrigation can be carried out in two ways, first by allowing the fluid to run in and directly out again, or by letting the bladder fill up before allowing it to flow out; to me the latter is the ideal.

In irrigation of the bladder we may use first normal saline solution, permanganate of potassium 20 grains to quart, boric acid, saturated solution, or bichloride, 1-50000. One irrigation of one quart a day is sufficient unless the case is severe. The best method is to use a fountain syringe of whatever pattern, with either glass or rubber catheter, preferably glass, for it is easily sterilized. Allow the solution to flow slowly in till the patient complains of fullness and discomfort; then we may feel sure that the fluid has distended the bladder walls and has come in contact with all parts, then allow it to flow out, continue this and at the end allow a little of the solution to remain in the bladder, directing that it be passed in an hour. Nitrate of silver solution, one to five per cent., also ichthyol solution, one to five per cent., may be used by irrigation, but normal saline should follow the stronger silver solutions.

In these old chronic cases, after a few irrigations you will notice the increased capacity of the bladder and irritability lessened. In the ulcerated variety, or where certain areas are affected, we can by the use of the Kelly speculum or cystoscope, with a good light, expose them to view, and make direct application to them, taking care only to apply to diseased areas; for this treatment nitrate of silver is best, using it from one to twenty per cent., according to the case.

Some advise injections into bladder by long syringe in stronger and smaller quantities than by irrigation, and allowing them to remain, ichthyol, ten per cent.; iodoform, ten per cent. emulsion, and nitrate of silver, but we have no direct proof that they come in contact with all the diseased portions, and so I think irrigation and local application are practicable. Yet in the tubercular variety we can not help but know that the stronger solutions will accomplish more in a short time than irrigation would.

Surgery offers some means of relief where other methods have failed. In the first place put bladder at rest by making a vesico-vaginal fistula; irrigate the bladder daily afterwards through the urethra until cured, then close up the fistula. The objection to this is the constant dribbling of urine out by way of vagina, causing inconvenience and irritation.

Curettage of the bladder may be accomplished through a cystoscope; this is indicated where we have tubercular or indolent ulcers. Phosphatic deposits may be present and cause the condition, but they can usually be broken up by the finger through the urethra into the bladder.—*American Practitioner and News.*

